# Qualitative and Quantitative Evaluation of Chemical Constituents from Shuanghuanglian Injection Using Nuclear Magnetic Resonance Spectroscopy

**DOI:** 10.1155/2022/7763207

**Published:** 2022-03-09

**Authors:** Ziyan Wang, Zuoyuan Wang, Miaomiao Jiang, Jing Yang, Qingfen Meng, Jianli Guan, Maoling Xu, Xin Chai

**Affiliations:** ^1^State Key Laboratory of Component-based Chinese Medicine, Tianjin Key Laboratory of TCM Chemistry and Analysis, Tianjin University of Traditional Chinese Medicine, Tianjin 301617, China; ^2^Henan Fusen Pharmaceutical Co.,Ltd., Henan 474450, China; ^3^Tianjin Medical University General Hospital, Tianjin 300052, China

## Abstract

By employing nuclear magnetic resonance (NMR), we implemented a chemical research on Shuanghuanglian injection (SHLI) and identified 17 components, including eight primary metabolites and nine secondary metabolites. Guided by the approach of network pharmacology, the potential activities were briefly predicted for seven primary metabolites except for formic acid, such as anti-inflammation, antioxidation, and cardiovascular protection. The focused primary metabolites were quantified by a proton nuclear magnetic resonance (^1^H-NMR) method, which was verified with good linearity and satisfactory precision, repeatability, stability, and accuracy (except for *myo*-inositol with mean recovery at 135.78%). Based on the successfully established method, seven primary metabolites were effectively quantified with a slight fluctuation in 20 batches of SHLIs. The average total content of these compounds was 6.85 mg/mL, accounting for 24.84% in total solid of SHLI. This research provides an alternative method for analysis of primary metabolites and contributes to the quality control of SHLI.

## 1. Introduction

Traditional Chinese medicine injections (TCMIs) are composed of active substances extracted from Chinese materia medica (CMM), which have been widely used in clinical application, such as the treatment of diseases in respiratory and cardiovascular system and cancer [1–4]. For instance, Kanglaite injection, a neutral oil extracted and isolated from coix seed, has been filed in application for investigational new drug to the Food and Drug Administration, which has shown an encouraging antineoplastic activity and a well-tolerated safety profile in phase II clinical development [5, 6]. However, the complex chemical constituents are still a bottleneck for exploration of in-depth action mechanisms and clinical application for most TCMIs that are required to perform more rigorous chemical investigations and quality controls. Especially, with high polarity and no ultraviolet absorption, the analysis of primary metabolites is still a challenge.

As a typical TCMI for treatment of symptoms such as acute upper respiratory tract infection, fever, and pneumonia [7, 8], Shuanghuanglian injection (SHLI), composed of the extract of *Lonicerae Japonicae Flos*, *Forsythiae Fructus*, and *Scutellariae Radix*, has the function of heat-clearing and detoxifying, as well as dispelling wind and relieving exterior symptoms. Previous researches have shown that SHLI has antiviral, anti-inflammatory, antioxidant effects, and so on [9–11]. SHLI effectively alleviates acute lung injury caused by lipopolysaccharide in mice [11]. Gao et al. found that SHLI relieved the yeast-induced pyrexia in rats by regulating the disordered metabolism through a variety of metabolic pathways [8]. For studying chemical constituents of SHLI, it still leaves a large space to improve. Up to now, more than 120 components have been identified in Shuanghuanglian powder injection, including flavonoids, phenolic acids, iridoid glycosides, and phenylethanoid glycosides [12–14]. Much attention was paid to the secondary metabolites [15, 16], while it was extremely limited on clarification of the primary metabolites, which are considered to perform helpful pharmacological activities.

With advantages of high-speed, nonselective, free of standard, and no complicated derivatization, nuclear magnetic resonance (NMR) is always employed to elucidate the structures of chemical compounds and perform quantitative analysis, especially for the primary metabolites [17]. Thus, quantitative nuclear magnetic resonance (qNMR) was widely applied in studying pharmaceuticals [18, 19], natural products [20], and metabolomics [21]. For example, terpene trilactones in *Ginkgo biloba* leaf extract were analyzed by quantitative ^1^H-NMR, whose result was generally consistent to the data determined by high-performance liquid chromatography [22]. By employing anthracene as internal standard, Hazekamp et al. rapidly measured the content of cannabinoids in *Cannabis sativa* with ^1^H-NMR method [23]. Noticeably, determination of the primary metabolites can be satisfactorily accomplished by quantitative ^1^H-NMR without derivatization [17, 24].

In our previous study, the secondary metabolites of SHLI were qualitatively and quantitatively illuminated by UHPLC/Q-Orbitrap-MS and UPLC-PDA [25]. In this case, we mainly focused on the study of the primary metabolites in SHLI by the quantitative ^1^H-NMR method. Eight primary metabolites and nine secondary metabolites were identified by ^1^H-NMR, ^13^C-NMR, and two-dimensional (2D) NMR spectra, including valine, glucose, fructose, mannose, sucrose, formic acid, succinic acid, and *myo*-inositol as primary metabolites, and four phenylpropanoids, three phenylethanoid glycosides, a flavone, and an iridoid as secondary metabolites. Focusing on the primary metabolites except for formic acid, the possible pharmacological activity and related pathways were briefly predicted by the network pharmacology. Subsequently, a quantitative ^1^H-NMR method was established in the light of the characteristic proton signals of the detected primary metabolites. Following the successful methodologic validation, seven primary metabolites were quantified in 20 batches of SHLIs, which accounted for 24.84% in total solid of SHLI. To the best of our knowledge, the quantitative ^1^H-NMR method has been innovatively applied for the chemical profiling of SHLI. The developed quantitative ^1^H-NMR will provide a reliable and rapid approach for quantifying the primary metabolites in SHLI, as well as an alternative method for quality control of SHLI.

## 2. Materials and Methods

### 2.1. Reagents and Materials

Twenty batches of SHLIs produced in 2018 and 2019 were obtained from Henan FuSen Pharmaceutical Co., Ltd. (Henan, China), which were numbered as B1–B20. Water used in the experiment was produced by a Millipore Milli-Q system (Milford, MA, USA). Neochlorogenic acid (P27A10L87091, ≥98.00%), cryptochlorogenic acid (P30A9L69104, ≥98.00%), isoforsythiaside A (Y14M6H1, ≥98.00%), forsythoside *E* (P23N7F25442, ≥98.00%), *L*-valine (H02J10Y91720, 99.00%), *D*-glucose (S14O10H99780, 99.00%), and *D*-fructose (J01J10R89818, 99.00%) were purchased from Shanghai Yuanye Bio-Technology Co., Ltd. (Shanghai, China). Chlorogenic acid (110753–201817, >96.80%), caffeic acid (110885–201703, >99.70%), baicalin (110715–201821, >95.40%), forsythoside A (111810–201707, >97.20%), and *myo*-inositol (190077–201501, ≥99.60%) were acquired from National Institutes for Food and Drug Control (Beijing, China). Secoxyloganin (DST190803-111, ≥98.00%) was purchased from Chengdu DeSiTe Biological Technology Co., Ltd. (Sichuan, China). *D*-mannose (*F*1523118, ≥98.00%) and sucrose (39030, 99.90%) were provided by Shanghai Aladdin Biological Technology Co., Ltd. (Shanghai, China). Succinic acid (BCBM0043V, ≥98.00%) was obtained from Sigma-Aldrich Inc. (St. Louis, MO, USA). 36.5% deuterium chloride was supplied by Shanghai Acmec Biochemical Technology Co., Ltd. (Shanghai, China). Deuteroxide (D_2_O) and sodium 3-trimethylsilyl propionate-2,2,3,3-*d*_4_ (TSP-*d*_4_) were acquired from Sigma-Aldrich Inc.

### 2.2. Sample Preparation

The precisely transferred SHLI (1 mL) was mixed with 1 mL D_2_O containing 0.2322 mM TSP-*d*_4_. D_2_O was used for the internal lock signal and TSP-*d*_4_ served as the internal standard with the chemical shift at *δ* 0.0. The mixed solution (0.5 mL) was transferred into a NMR tube (WG-5000, Wilmad, USA) for the further test.

SHLI (10 mL) was precisely transferred into 10 mL D_2_O containing 0.2322 mM TSP-*d*_*4*_, which was mixed as stock solution in a centrifuge tube. As reference standards, 17 compounds were appropriately weighed and, respectively, dissolved in 0.6 mL stock solution, which was, respectively, transferred into NMR tubes for verifying the identified compounds.

The samples of the tested SHLIs were frozen at –80°C for 12 h and freeze-dried in a vacuum freeze-dryer (FDU-2110, EYELA, Tokyo, Japan) for 24 h, and then the lyophilized powder was obtained.

### 2.3. Standard Solution Preparation

The 36.5% deuterium chloride was diluted by 100-fold using D_2_O. An equal volume of water was added into D_2_O containing 0.2322 mM TSP-*d*_4_, which was subsequently adjusted by 0.365% deuterium chloride to reach pH 5.38–5.48 as solvent for preparing standard solution.

*L*-valine, *D*-glucose, *D*-fructose, *D*-mannose, sucrose, succinic acid, and *myo*-inositol were accurately weighed and, respectively, dissolved in a volumetric flask to reach concentrations at 0.1320, 6.9180, 8.9108, 0.6840, 2.1320, 0.0961, and 3.6924 mg/mL.

### 2.4. NMR Spectroscopy

All the NMR spectra were acquired at 298 K on a 600 MHz BRUKER AVANCE III spectrometer (Bruker, Switzerland) equipped with a cryoprobe. All pulse sequences were undertaken from the Bruker pulse program library. The standard *noesygppr1d* was employed as a water peak suppression pulse sequence. The 90° pulse width was adjusted to 9 *μ*s for each sample. A total of 65536 data points were collected via 16 scans under conditions of detective frequency at 600.20 MHz, spectrum width (SW) at 20.0253 ppm, central position (O_1_) at 4.701 ppm, and a relaxation delay of 5 s. In order to accurately assign the proton signals of the studied compounds, ^13^C-NMR, ^1^H-^1^H correlation spectroscopy (^1^H-^1^H COSY), heteronuclear single quantum coherence (HSQC), and heteronuclear multiple bond correlation (HMBC) spectra were recorded for the tested samples. From the tested compounds and TSP-*d*_*4*_, spin-lattice relaxation time (T_1_) values of the quantified protons were measured using a classical inversion recovery pulse sequence with 20 relaxation delays (*τ*) ranging from 0.001 to 20 s.

### 2.5. Predictions of Targets and Pathways, and KEGG Pathway Enrichment Analysis

Chemical structures of the seven primary metabolites were acquired from the PubChem database (https://pubchem.ncbi.nlm.nih.gov/) [26]. Potential molecular targets of the interesting compounds were predicted in the SwissTargetPrediction database (http://www.swisstargetprediction.ch/) [27]. The Kyoto Encyclopedia of Genes and Genomes (KEGG) pathway enrichment was performed by the Database for Annotation, Visualization and Integrated Discovery 6.8 (DAVID) (https://david.ncifcrf.gov/summary.jsp/). The potential activities were summarized via pathway analysis of the KEGG (https://www.genome.jp/kegg/). Origin 9.6 software was employed to construct the network of ingredients-targets-pathways-activities.

### 2.6. Quantification of Seven Primary Metabolites

The original NMR data were processed by MestReNova 6.1.0 (Mestrelab Research S. L., Santiago de Compostela, Spain) with automatic correction of phase and baseline. Because the intensity of signal is positively correlated with its contributing number of protons detected in ^1^H-NMR, the integral areas of quantitative signals were used to determine the content of the tested compounds according to the following equation:

(1) $$\begin{array}{c} C_{X}=\frac{M_{X}\cdot N_{TSP}\cdot C_{TSP}\cdot A_{X}}{1000\cdot N_{X}\cdot A_{TSP}}. \end{array}$$

*X*, the different compounds tested in this study; *C*_*X*_, the mass concentration of the tested compounds (mg/mL); *M*_*X*_, molar mass of the tested compounds; *C*_*TSP*_, molarity of TSP-*d*_4_ (mM); *N*_*TSP*_ and *N*_*X*_, the proton numbers per mole TSP-*d*_4_ and the tested compounds, respectively; *A*_*TSP*_ and *A*_*X*_, the peak areas of quantitative protons of TSP-*d*_4_ and the tested compounds, respectively.

### 2.7. Statistical Analysis

The parallel coordinate, box, and double-Y plots were plotted using Origin 9.6 software (OriginLab, Northampton, MA, USA).

## 3. Results and Discussion

### 3.1. Proton Signal Assignments and Chemical Identification

SHLI with the features of multicomponents, multitargets, and complex mechanisms has shown great therapeutic advantages for upper respiratory tract infection. However, clarification of most chemical materials associated with the pharmacological activity of SHLI still remains to be enriched.

**Figure 1:**
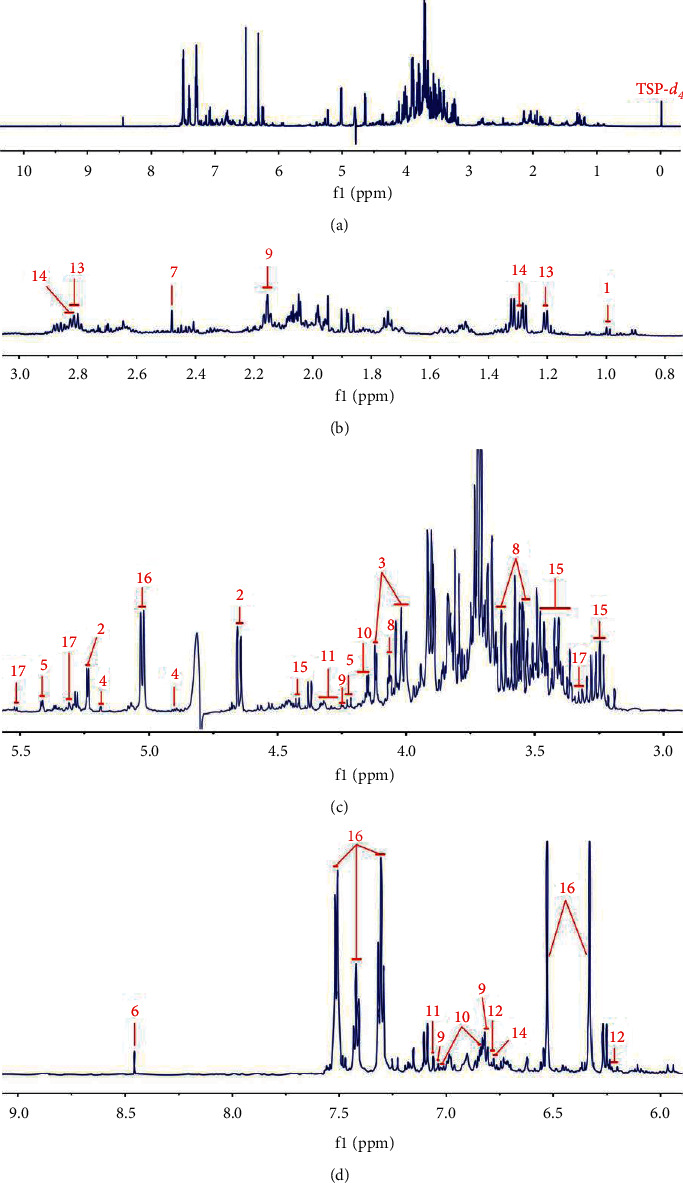
Representative ^1^H-NMR spectrum of SHLI from *δ* 0.0 to 10.0 (a), enlarged spectrum between *δ* 0.8 and 3.0 (b), from *δ* 3.0 to 5.5 (c), and in the range of *δ* 6.0 and 9.0 (d). TSP-*d*_4_ as an internal standard with a chemical shift at 0.0 ppm. Characteristic signal peaks of 17 metabolites: 1 valine; 2 glucose; 3 fructose; 4 mannose; 5 sucrose; 6 formic acid; 7 succinic acid; 8 *myo*-inositol; 9 chlorogenic acid; 10 neochlorogenic acid; 11 cryptochlorogenic acid; 12 caffeic acid; 13 forsythoside A; 14 isoforsythiaside A; 15 forsythoside E; 16 baicalin; 17 secoxyloganin.

**Figure 2:**
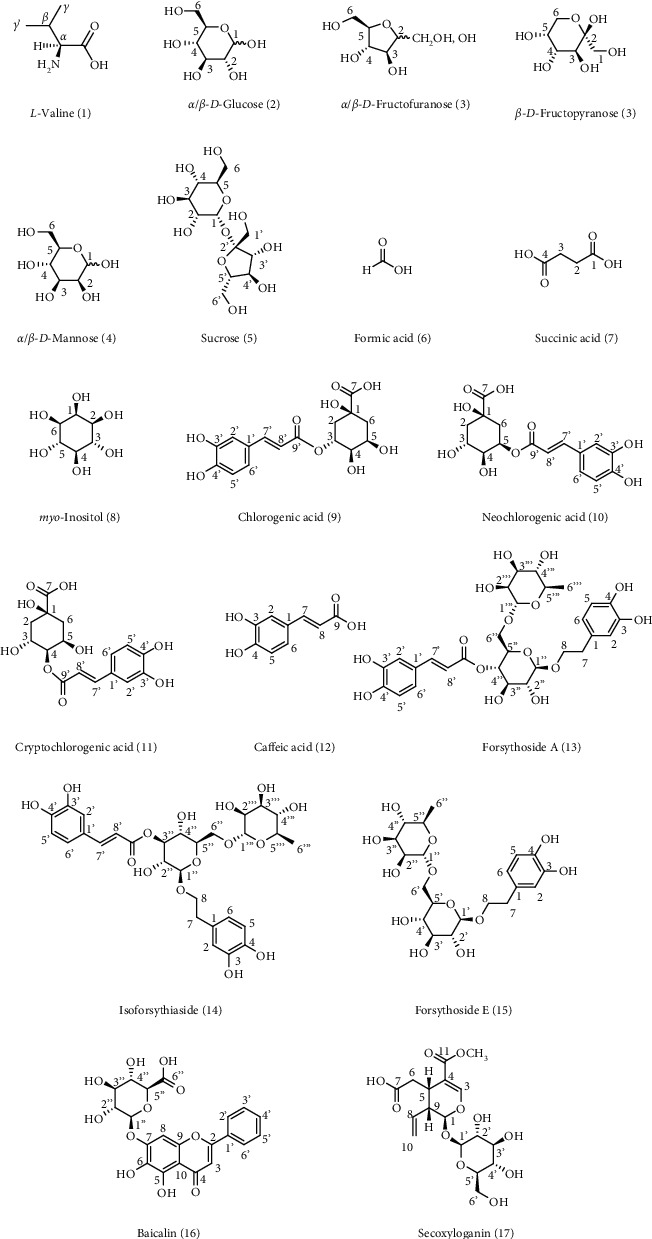
Chemical structures of 17 components in SHLI.

**Figure 3:**
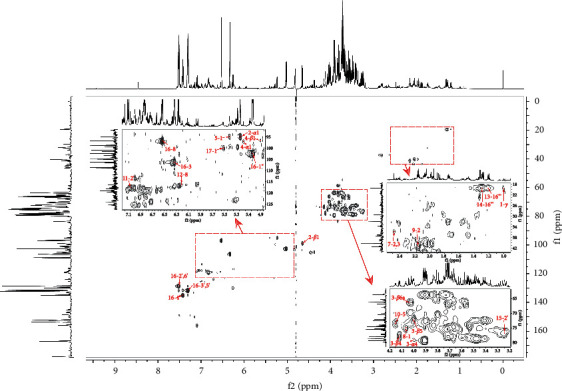
The key HSQC correlations of the identified components in HSQC spectrum of SHLI.

In our study, by employing NMR method, 17 metabolites were identified from SHLI, including eight primary metabolites and nine secondary metabolites. Representative ^1^H-NMR spectra of SHLI are shown in Figure 1, and ^13^C-NMR and 2D NMR spectra are displayed in Figures S1 and S2. The primary metabolites identified in SHLI included an amino acid (valine), two organic acids (succinic acid and formic acid), three monosaccharides (glucose, fructose, and mannose), a disaccharide (sucrose), and a cycloparaffin (*myo*-inositol). The secondary metabolites were elucidated as four phenylpropanoids (chlorogenic acid, neochlorogenic acid, cryptochlorogenic acid, and caffeic acid), three phenylethanoid glycosides (forsythosides A and E, and isoforsythiaside A), a flavone (baicalin), and an iridoid (secoxyloganin). Combined with ^13^C-NMR and 2D NMR spectra, the obtained protons signals were assigned to identify these metabolites. Due to the complexity and abundance discrepancy of the detected constituents in SHLI, part of the signals of protons inevitably overlapped, or the responses were low. Therefore, it is challenging to assign signals of these constituents. By the obtained spectra and published results, the signal assignments were performed as possible as we can for the characterized compounds, which are listed in Table 1. The chemical structures are displayed in Figure 2. The key HSQC correlations of the focused compounds are shown in Figure 3.

Taking *myo*-inositol as an example, TSP-*d*_4_ (0.1161 mM) in D_2_O provided a reference signal with a chemical shift at *δ* 0.0. Characteristic peaks of protons were available in the high field of ^1^H-NMR spectrum. Four doublets of doublets were, respectively, detected at 4.06, 3.63, 3.54, and 3.28 ppm, conducing to exploration of the six proton signals. ^13^C-NMR and 2D spectra were employed to assist clarification of structure. Mutual coupling constants combining with ^1^H-^1^H COSY revealed the relative positions of the protons. In accordance with the proton signals, the associated carbon signals at 75.1, 74.1, 75.3, and 77.3 ppm were confirmed by HSQC and HMBC. The proton and carbon signals were assigned for *myo*-inositol, which was finally verified by the spectra of standard substance and published result [36].

### 3.2. Prediction of Potential Targets and Action Pathways of Seven Primary Metabolites from SHLI

**Figure 4:**
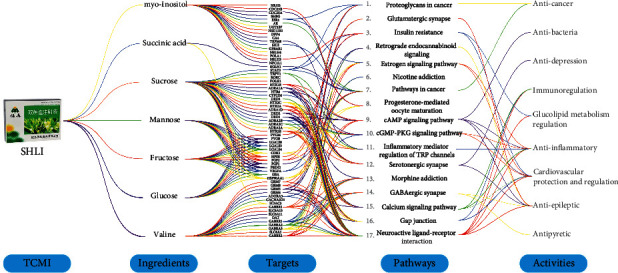
Parallel coordinate plot for the ingredients-targets-pathways-activities network of the focused primary metabolites from SHLI.

Through qualitative analysis, 17 chemical components were identified from SHLI. In this study, we paid more attention to the primary metabolites, whose potential pharmacological activities except for formic acid were briefly predicted with guidance of the network pharmacology. Chemical structures of the focused primary metabolites were obtained from PubChem database, which were imported for prediction of the potential targets by the reverse pharmacophore matching method in SwissTargetPrediction database. Using the DAVID database, the KEGG pathways associated with the targets were identified with human as a limited species. Enrichment analysis of the KEGG revealed the possible pharmacological activities and the targeted pathways involved. Successfully, an ingredients-targets-pathways-activities network was framed for seven primary metabolites, including valine, glucose, fructose, mannose, sucrose, succinic acid, and *myo*-inositol, which is shown in Figure 4.

Based on seven primary metabolites, 105 targets were collected from the database. After getting rid of 40 repetitive genes, only 65 targets were employed for pathway prediction. Enrichment of seventeen KEGG pathways for the predicted targets was implemented by the DAVID database. Pathway analysis in KEGG indicated that these focused primary metabolites may participate in anti-inflammatory effect by activating multiple targets such as TRPM8, TRPV1, HTR2B, and HTR2C, as well as pathways, including inflammatory mediator regulation of TRP channels, cGMP-PKG signaling pathway, and cAMP signaling pathway, which are also involved in anti-bacteria, immunoregulation, cardiovascular protection and regulation, and so on.

### 3.3. Methodological Validation of Seven Primary Metabolites from SHLI by Quantitative ^1^H-NMR Analysis

As an important acquisition parameter that affects the precision and accuracy of the quantitative result in ^1^H-NMR analysis, the relaxation delay value should be long enough to ensure complete relaxation for all the selected protons, which is set at least five times the longest T_1_ of the quantified protons [24]. In this study, the T_1_ values of the characteristic signals of protons for the targeted components were measured by inversion recovery pulse sequence experiment (Bruker pulprog: *t1irpr*). By taking the balance between the reliability of method and the analytical efficiency into account, the relaxation delay was set to five seconds in this study.

Based on the optimized NMR parameters, the internal reference standard method was preferentially chosen for quantitative analysis of the focused compounds from SHLI. As an internal reference standard, TSP-*d*_4_ possesses good water-solubility and stable properties, whose signal is a sharp single peak at chemical shift *δ* 0.0. Therefore, TSP-*d*_4_ was employed as a reliable reference for quantitative analysis of the primary metabolites, conducing to good solubility in SHLI and nonoverlapping of signal peaks with the tested compounds.

The signals of quantitative protons were assigned for seven primary metabolites and listed in Table 2. Notably, due to the existence of the different configurations for glucose, fructose, and mannose in SHLI, the quantitative result was expressed as the total content of the different configurations. Taking glucose as an example, *α* and *β* configurations were present in SHLI at an approximate ratio of 1 : 1.6. The two doublets at 5.24 and 4.65 ppm were selected as the characteristic signals of anomeric protons, which were simultaneously employed for quantification of glucose.

Methodological validation was subsequently performed for the quantitative ^1^H-NMR method, whose detailed result is shown in Table 3. The calibration curves were constructed by plotting the given concentrations of the standard solution (*x*) versus the average integral areas of the peaks for quantitative protons (*y*). Good linear relationships of seven primary metabolites (*r* values above 0.9997) were achieved. The RSD values of both intraday and interday precision were below 1.3%. The RSD values of repeatability and stability were 0.3%–2.7% and 0.3%–1.2%, respectively. Additionally, the average recoveries for the investigated metabolites ranged from 93.96% to 105.93% except for *myo*-inositol with 135.78%. The approved method was subsequently applied for determination of the focused primary metabolites in SHLI.

### 3.4. Quantification of Seven Primary Metabolites in Different Batches of SHLIs

**Figure 5:**
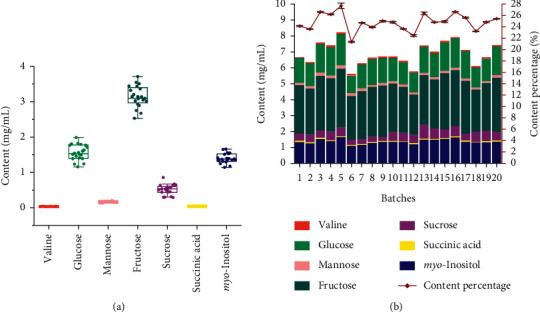
Boxplot for the quantitative analysis (a) and double-Y plot for the total content and content percentage (b) of seven primary metabolites in 20 batches SHLIs (*n* = 3).

Using the established ^1^H-NMR method, seven primary metabolites were quantified, whose content showed a slight fluctuation in 20 batches of SHLIs. The content reached 0.0288–0.0416 mg/mL for valine, 1.15–1.99 mg/mL for glucose, 2.53–3.71 mg/mL for fructose, 0.130–0.200 mg/mL for mannose, 0.292–0.854 mg/mL for sucrose, 0.0257–0.0447 mg/mL for succinic acid, and 1.14–1.66 mg/mL for *myo*-inositol, respectively. Specific results are listed in Table S1. The average content of seven compounds was 0.0338, 1.56, 3.14, 0.165, 0.513, 0.0356, and 1.40 mg/mL, individually. A detailed description is shown by the boxplot in Figure 5(a), which reflects the dispersion of content for the focused primary metabolites in 20 batches of SHLIs. The content of valine, mannose, and succinic acid was extremely low. Reversely, fructose, glucose, *myo*-inositol, and sucrose were proved to be high abundant.

In order to clarify the total content of these seven primary metabolites in the total solid of SHLI, the freeze-dried powder of 20 batches SHLIs was determined in the range of 25.2 and 30.7 mg/mL with the RSD value of 5.1%. The total content of the tested compounds in SHLI ranged from 5.58 to 8.18 mg/mL, the mean content of which was 6.85 mg/mL, accounting for 24.84% in total solid of SHLI. The results are displayed in Figure 5(b). In this study, the quantitative analysis of these seven compounds enriches the chemical composition research of SHLI and provides a feasible method for the quality evaluation of the primary metabolites from SHLI.

## 4. Conclusions

In conclusion, by the aid of NMR approach, 17 chemical compounds in SHLI were identified, including eight primary metabolites and nine secondary metabolites. The ingredients-targets-pathways-activities network was preliminarily predicted for seven primary metabolites by the help of network pharmacology. Moreover, a quantitative ^1^H-NMR method was established with good linearity, precision, repeatability, and accuracy, which paved the way for simultaneous quantification of the focused primary metabolites. The established method provides an alternative for quality evaluation of the primary metabolites, which conduces to improvement of SHLI quality.

## Figures and Tables

**Table 1:** The assignments of chemical shifts for 17 components by ^1^H-NMR and^13^C-NMR spectra of SHLI (H_2_O : D_2_O = 1 : 1).

Metabolites	*δ * ^1^H and its assignment	*δ * ^13^C and its assignment
Valine [28]	3.61 (d, *J* = 4.8 Hz, *α*-H), 2.28 (m, *β*-H), 0.99 (d, *J* = 7.2 Hz, *γ*-H_3_), 0.91 (d, *J* = 7.2 Hz, *γ′*-H_3_)	63.4 (*α*-C), 32.0 (*β*-C), 20.8 (*γ*-C), 19.5 (*γ′*-C), − (C=O)
Glucose [29,30]	5.24 (d, *J* = 3.6 Hz, *α*H-1), 3.54 (dd, *J* = 3.0, 10.2 Hz, *α*H-2), 3.70–3.78 (m, *α*H-3, *α*H-6b, *β*H-6b), 3.39–3.43 (m, *α*H-4, *β*H-4), 3.83–3.86 (m, *α*H-5, *α*H-6a), 4.65 (d, *J* = 7.8 Hz, *β*H-1), 3.25 (dd, *J* = 8.4, 9.6 Hz, *β*H-2), 3.45–3.51 (m, *β*H-3, *β*H-5), 3.90 (m, *β*H-6a)	95.0 (*α*C-1), 74.3 (*α*C-2), 75.7 (*α*C-3), 72.6 (*α*C-4), 74.4 (*α*C-5), 63.5 (*α*C-6), 98.8 (*β*C-1), 77.1 (*β*C-2), 78.7 (*β*C-3), 72.5 (*β*C-4), 78.8 (*β*C-5), 63.7 (*β*C-6)
Fructose [31, 32]	3.66–3.68 (m, *α*-*f* H-1), 4.12 (m, *α*-*f* H-3), 4.00 (m, *α*-*f* H-4, *β*-*p* H-5), 4.06 (m, *α*-*f* H-5), 3.81–3.85 (m, *α*-*f* H-6a), 3.68–3.73 (m, *α*-*f* H-6b), 3.60 (d, *J* = 12.0 Hz, *β*-*f* H-1a), 3.56 (d, *J* = 12.0 Hz, *β*-*f* H-1b), 4.12 (m, *β*-*f* H-3, H-4), 3.61–3.83 (m, *β*-*f* H-5), 3.66–3.82 (m, *β*-*f* H-6), 3.72 (d, *J* = 12.0 Hz, *β*-*p* H-1a), 3.57 (d, *J* = 12.0 Hz, *β*-*p* H-1b), 3.80 (d, *J* = 10.2 Hz, *β*-*p* H-3), 3.90 (dd, *J* = 3.0, 10.2 Hz, *β*-*p* H-4), 4.03 (brd, *J* = 12.6 Hz, *β*-*p* H-6a), 3.71 (brd, *β*-*p* H-6b)	65.8 (*α*-*f* C-1), − (*α*-*f* C-2), − (*α*-*f* C-3), 79.0 (*α*-*f* C-4), 84.2 (*α*-*f* C-5), 65.6 (*α*-*f* C-6), 65.3 (*β*-*f* C-1), 104.4 (*β*-*f* C-2), 78.3 (*β*-*f* C-3), 77.3 (*β*-*f* C-4), 83.6 (*β*-*f* C-5), 64.1 (*β*-*f* C-6), 66.8 (*β*-*p* C-1), 101.0 (*β*-*p* C-2), 70.5 (*β*-*p* C-3), 72.6 (*β*-*p* C-4), 72.1 (*β*-*p* C-5), 66.2 (*β*-*p* C-6)
Mannose [33]	5.19 (d, *J* = 1.2 Hz, *α*H-1), 3.89–3.95 (m, *α*H-2, *β*H-2, *β*H-6a), 3.81–3.87 (m, *α*H-3, *α*H-5, *α*H-6a), 3.64–3.68 (m, *α*H-4, *β*H-3), 3.71–3.78 (m, *α*H-6b, *β*H-6b), 4.90 (brs, *β*H-1), 3.56–3.59 (m, *β*H-4), 3.36–3.40 (m, *β*H-5)	96.9 (*α*C-1), 73.6 (*α*C-2), 73.1 (*α*C-3), − (*α*C-4), 75.3 (*α*C-5), − (*α*C-6), 96.2 (*β*C-1), 74.2 (*β*C-2), 76.0 (*β*C-3), − (*β*C-4), 79.1 (*β*C-5), − (*β*C-6)
Sucrose [34]	5.42 (d, *J* = 3.6 Hz, H-1), 3.57 (m, H-2), 3.77 (m, H-3), 3.48 (m, H-4), 3.81–3.86 (m, H-5, H-6, H-6′), 3.68 (s, H-1′), 4.22 (d, *J* = 8.4 Hz, H-3′), 4.06 (m, H-4′), 3.90 (m, H-5′)	95.0 (C-1), − (C-2), 75.5 (C-3), 72.1 (C-4), 75.3 (C-5), 63.0 (C-6), 64.2 (C-1′), 106.5 (C-2′), − (C-3′), 76.9 (C-4′), 84.2 (C-5′), 65.3 (C-6′)
Formic acid [35]	8.46 (s, H-1)	− (C-1)
Succinic acid [35]	2.48 (s, H-2, H-3)	− (C-1, C-4), − (C-2, C-3)
*myo*-inositol [36]	4.06 (dd, *J* = 3.0, 3.0 Hz, H-1), 3.54 (dd, *J* = 3.0, 9.6 Hz, H-2, H-6), 3.63 (dd, *J* = 9.6, 9.6 Hz, H-3, H-5), 3.28 (dd, *J* = 9.6, 9.6 Hz, H-4)	75.1 (C-1), 74.1(C-2, C-6), 75.3 (C-3, C-5), 77.3 (C-4)
Chlorogenic acid [37]	2.16 (m, H-2), 5.27 (m, H-3), 3.85 (dd, *J* = 3.0, 9.0 Hz, H-4), 4.25 (m, H-5), 2.02 (m, H-6), 7.04 (d, *J* = 1.8 Hz, H-2′), 6.81 (d, *J* = 8.4 Hz, H-5′), 6.97 (dd, *J* = 1.8, 8.4 Hz, H-6′), 7.55 (d, *J* = 15.6 Hz, H-7′), 6.25 (d, *J* = 15.6 Hz, H-8′)	—
Neochlorogenic acid [38]	1.93 (m, H-2), 5.36 (m, H-3), 3.96 (m, H-4), 4.16 (m, H-5), 2.04–2.14 (m, H-6), 7.09 (brs, H-2′), 6.84 (d, *J* = 7.8 Hz, H-5′), 7.02 (dd, *J* = 1.8, 7.8 Hz, H-6′), 7.55 (d, *J* = 16.2 Hz, H-7′), 6.34 (d, *J* = 16.2 Hz, H-8′)	—
Cryptochlorogenic acid [38]	2.03 (m, H-2), 4.30 (m, H-3), 3.96 (m, H-4), 4.90 (*m*, H-5), 2.16 (m, H-6), 7.06 (d, *J* = 1.8 Hz, H-2′), 6.83 (d, *J* = 8.4 Hz, H-5′), 6.99 (dd, *J* = 1.8, 8.4 Hz, H-6′), 7.55 (d, *J* = 16.2 Hz, H-7′), 6.33 (d, *J* = 16.2 Hz, H-8′)	—
Caffeic acid [31]	7.00 (d, *J* = 1.8 Hz, H-2), 6.78 (d, *J* = 7.8 Hz, H-5), 6.92 (dd, *J* = 1.8, 8.4 Hz, H-6), 7.30 (d, *J* = 16.2 Hz, H-7), 6.22 (d, *J* = 16.2 Hz, H-8)	—
Forsythoside A [39]	6.82 (brs, H-2), 6.78 (d, *J* = 7.2 Hz, H-5), 6.72 (brd, *J* = 7.2 Hz, H-6), 2.81 (t, *J* = 7.8 Hz, H-7), 4.02 (m, overlapped, H-8a, H-6″a), 3.63–3.83 (m, overlapped, H-8b, H-6″b, H-3‴, H-5‴), 7.00 (d, *J* = 1.8 Hz, H-2′), 6.84 (d, *J* = 8.4 Hz, H-5′), 6.92 (dd, *J* = 2.4, 8.4 Hz, H-6′), 7.50 (d, *J* = 16.2 Hz, H-7′), 6.22 (d, *J* = 16.2 Hz, H-8′), 4.44 (d, *J* = 7.2 Hz, H-1″), 3.34–3.42 (m, overlapped, H-2″, H-4‴), 3.47 (m, H-3″, H-5″), 4.90 (dd, *J* = 9.6, 9.6 Hz, H-4″), 4.66 (brs, H-1‴), 3.84 (brs, H-2‴), 1.21 (d, *J* = 6.6 Hz, H-6‴)	—
Isoforsythiaside A [40]	6.82 (brs, H-2), 6.77 (d, *J* = 7.8 Hz, H-5), 6.72 (dd, *J* = 1.8, 7.8 Hz, H-6), 2.82 (t, *J* = 7.8 Hz, H-7), 4.00–4.07 (m, overlapped, H-8a, H-6″a), 3.64–3.83 (*m*, overlapped, H-8b, H-6″b, H-3‴, H-5‴), 6.98 (brs, H-2′), 6.83 (d, *J* = 8.4 Hz, H-5′), 6.92 (brd, *J* = 9.0 Hz, H-6′), 7.48 (d, *J* = 16.2 Hz, H-7′), 6.25 (d, *J* = 16.2 Hz, H-8′), 4.57 (d, *J* = 7.8 Hz, H-1″), 3.44–3.48 (m, overlapped, H-2″, H-4‴), 5.04 (m, H-3″), 3.51 (m, H-4″, H-5″), − (H-1‴), 3.83 (d, *J* = 1.8 Hz, H-2‴), 1.29 (d, *J* = 6.6 Hz, H-6‴)	—
Forsythoside *E* [41]	6.81 (d, *J* = 1.2 Hz, H-2), 6.83 (d, *J* = 7.8 Hz, H-5), 6.71 (dd, *J* = 1.8, 7.8 Hz, H-6), 2.80 (t, *J* = 7.2 Hz, H-7), 3.97–4.04 (m, overlapped, H-8a, H-6′a), 3.68–3.83 (m, overlapped, H-8b, H-6′b, H-3″, H-5″), 4.42 (d, *J* = 7.8 Hz, H-1′), 3.25 (dd, *J* = 8.4, 9.6 Hz, H-2′), 3.51–3.53 (m, overlapped, H-3′-H-5′), − (H-1″), 3.84 (brs, H-2″), 3.36–3.48 (m, H-4″), 1.28 (d, *J* = 6.0 Hz, H-6″)	−
Baicalin [42]	6.33 (s, H-3), 6.53 (s, H-8), 7.51 (d, *J* = 7.8 Hz, H-2′, H-6′), 7.30 (t, *J* = 7.8 Hz, H-3′, H-5′), 7.42 (t, *J* = 7.8 Hz, H-4′), 5.03 (d, *J* = 7.2 Hz,H-1″), 3.72 (m, H-2″), 3.71 (m, H-3″), 3.66 (m, H-4″), 3.91 (m, H-5″)	167.2 (C-2), 106.4 (C-3), 185.5 (C-4), 148.5 (C-5), 135.1 (C-6), 153.8 (C-7), 96.9 (C-8), 152.6 (C-9), 108.8 (C-10), 132.2 (C-1′), 128.7 (C-2′, C-6′), 131.7 (C-3′, C-5′), 135.1 (C-4′), 102.6 (C-1″), 75.4 (C-2″), 78.0 (C-3″), 74.7 (C-4″), 79.4 (C-5″), 178.0 (C-6″)
Secoxyloganin [43]	5.52 (d, *J* = 5.4 Hz, H-1), 7.53 (brs, H-3), 2.76 (m, H-5, H-2′), 2.30 (dd, *J* = 8.4, 15.6 Hz, H-6a), 2.53–2.60 (m, H-6b, H-9), 5.70 (ddd, *J* = 9.6, 10.2, 17.4 Hz, H-8), 5.32 (m, H-10), 4.65 (d, *J* = 7.8 Hz, H-1′), 3.33 (m, H-3′), 3.21 (m, H-4′), 3.41 (m, H-5′), 3.93 (m, H-6a′), 3.41 (m, H-6b′), 3.72 (s, –OCH_3_)	—

Singlet (s), doublet (d), triplet (t), doublet of doublets (dd), doublet of doublets of doublets (ddd), quintet (q), multiplet (m), broad doublet (brd), broad singlet (brs), furanose (f), pyranose (p), proton signal peaks were overlaid in ^1^H-NMR and carbon signal responses were too low or overlapped in ^13^C-NMR (−).

**Table 2:** Chemical shift and signals assignment of quantitative protons for seven primary metabolites.

Primary metabolites	Chemical shift and signals assignment of the quantitative protons
Valine	0.99 (*γ*-H_3_)
Glucose	5.24 (*α*H-1), 4.65 (*β*H-1)
Fructose	4.12 (*α*-*f* H-3, *β*-*f* H-3, H-4), 4.03 (*β*-*p* H-6a), 4.00 (*β*-*p* H-5, *α*-*f* H-4)
Mannose	5.19 (*α*H-1), 4.90 (*β*H-1)
Sucrose	5.42 (H-1)
Succinic acid	2.48 (H-2, H-3)
*myo*-inositol	4.06 (H-1)

**Table 3:** Summary results of linear regression, LODs, LOQs, precision, repeatability, stability, and recovery for the primary metabolites in SHLI.

Primary metabolites	Linear regression			Precision RSD (%)		Repeatability (*n* = 6, RSD, %)	Stability (*n* = 6, RSD, %)	Recovery (*n* = 6, mean ± SD, %)
	Regression equation	Linear range (mg/mL)	Correlation coefficients (*r*)	Intraday	Interday			
Valine	*y* = 274.19*x* − 0.0613	0.0010–0.1320	0.9998	1.3	1.0	0.8	1.2	98.43 ± 1.55
Glucose	*y* = 60.268*x* − 2.3468	0.0540–6.9180	0.9998	0.6	0.5	1.9	0.3	100.94 ± 1.74
Fructose	*y* = 123.09*x* − 6.5731	0.0696–8.9108	0.9997	0.4	0.6	0.3	0.6	102.30 ± 1.52
Mannose	*y* = 53.151*x* + 0.3726	0.0053–0.6840	0.9999	1.1	1.3	2.7	0.7	105.93 ± 0.52
Sucrose	*y* = 31.521*x* − 0.1564	0.0167–2.1320	0.9998	0.4	0.5	1.1	0.5	93.96 ± 1.14
Succinic acid	*y* = 364.01*x* − 0.1605	0.0008–0.0961	0.9997	0.4	0.7	0.6	0.4	102.66 ± 1.69
*myo*-Inositol	*y* = 85.074*x* − 1.9043	0.0288–3.6924	0.9997	0.7	0.7	0.4	0.4	135.78 ± 1.21

## Data Availability

The data used to support the findings of this study are available from the corresponding author upon request.

## Supplementary Materials

Supplementary Materials Figure S1: ^13^C-NMR spectrum of SHLI. Figure S2: 2D NMR spectra of SHLI (a) ^1^H-^1^H COSY; (b) HSQC; (c) HMBC). Table S1: the quantified results of seven primary metabolites in 20 batches of SHLIs (*n* = 3, mg/mL).
