# Supplementary material for: Qualitative and Quantitative Evaluation of Chemical Constituents from Shuanghuanglian Injection Using Nuclear Magnetic Resonance Spectroscopy
Source: J Anal Methods Chem. 2022 Mar 9;2022:7763207. doi: 10.1155/2022/7763207 (PMC8926469; doi:10.1155/2022/7763207)
Supplement: Supplementary Materials — Figure S1: 13C-NMR spectrum of SHLI. Figure S2: 2D NMR spectra of SHLI (a) 1H-1H COSY; (b) HSQC; (c) HMBC). Table S1: the quantified results of seven primary metabolites in 20 batches of SHLIs (n = 3, mg/mL). [file 7763207.f1.docx]

**Supplementary Materials to:**

**Qualitative and quantitative evaluation of chemical constituents from shuanghuanglian injection using nuclear magnetic resonance spectroscopy**


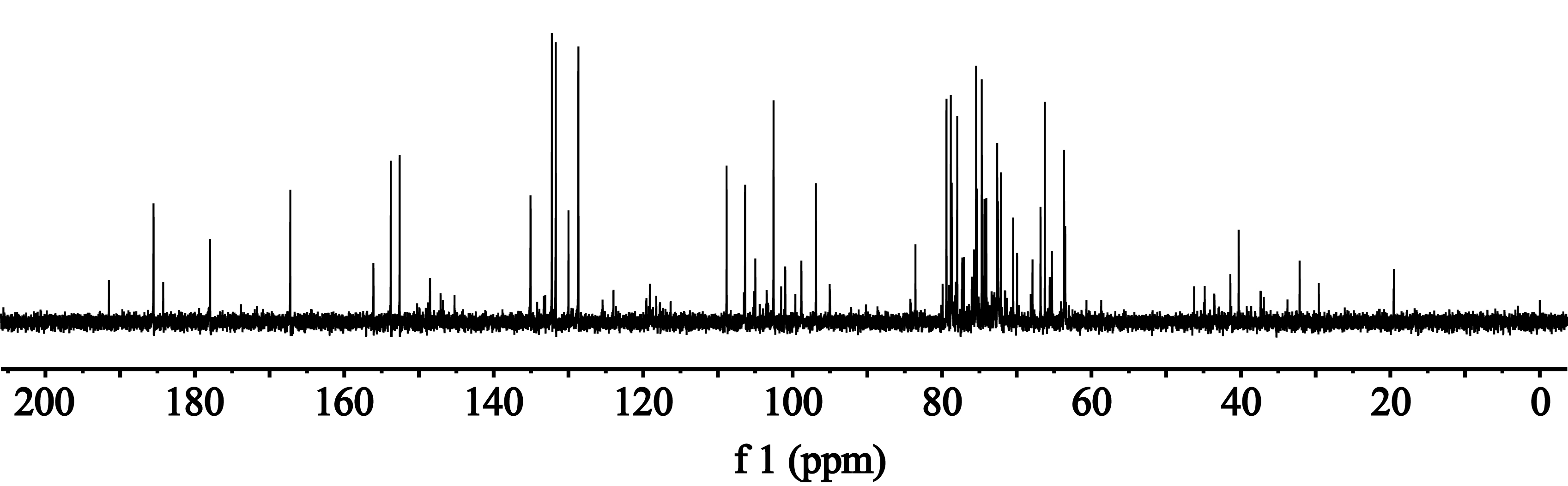


Figure S1: ^13^C-NMR spectrum of SHLI.


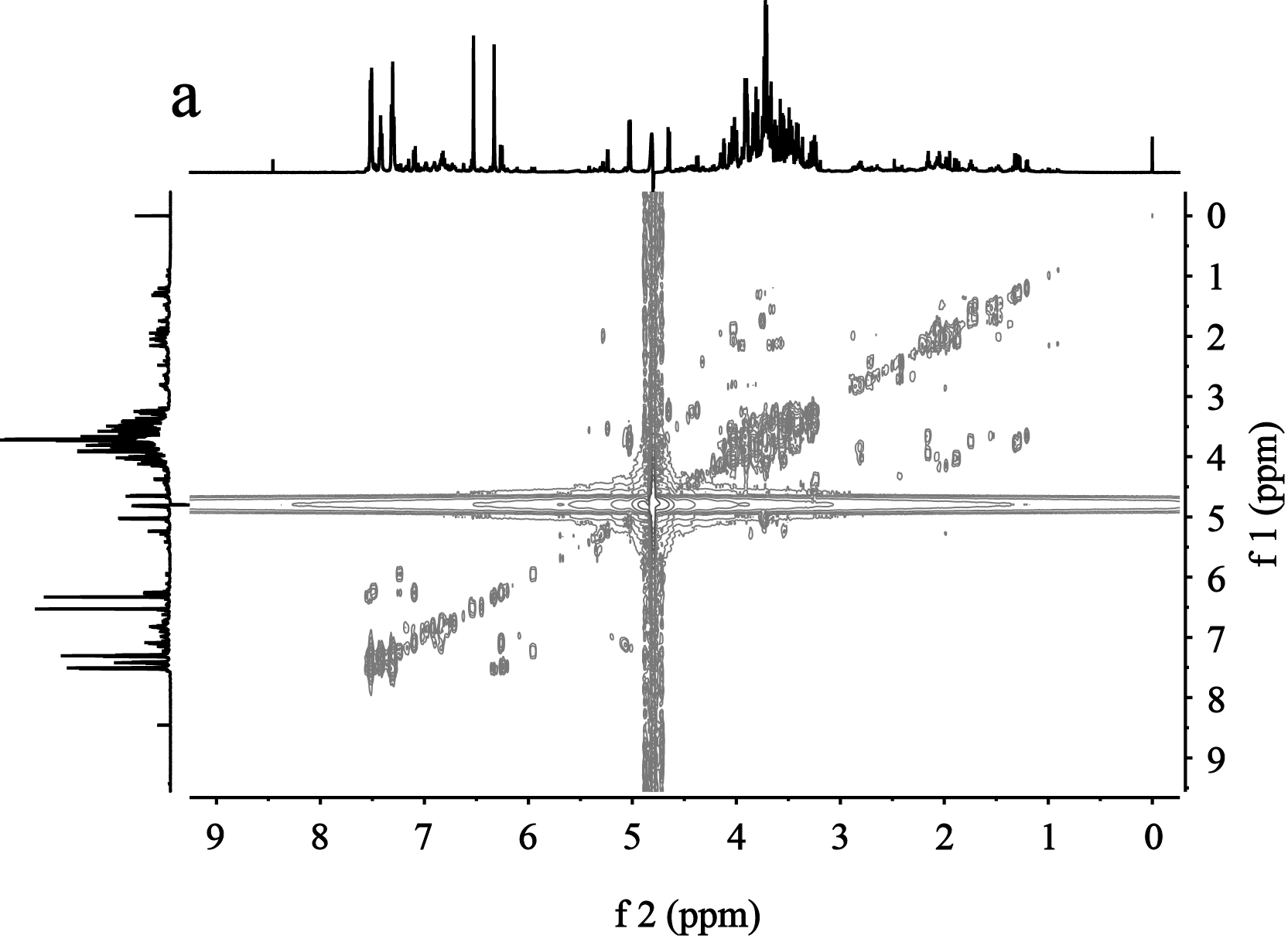


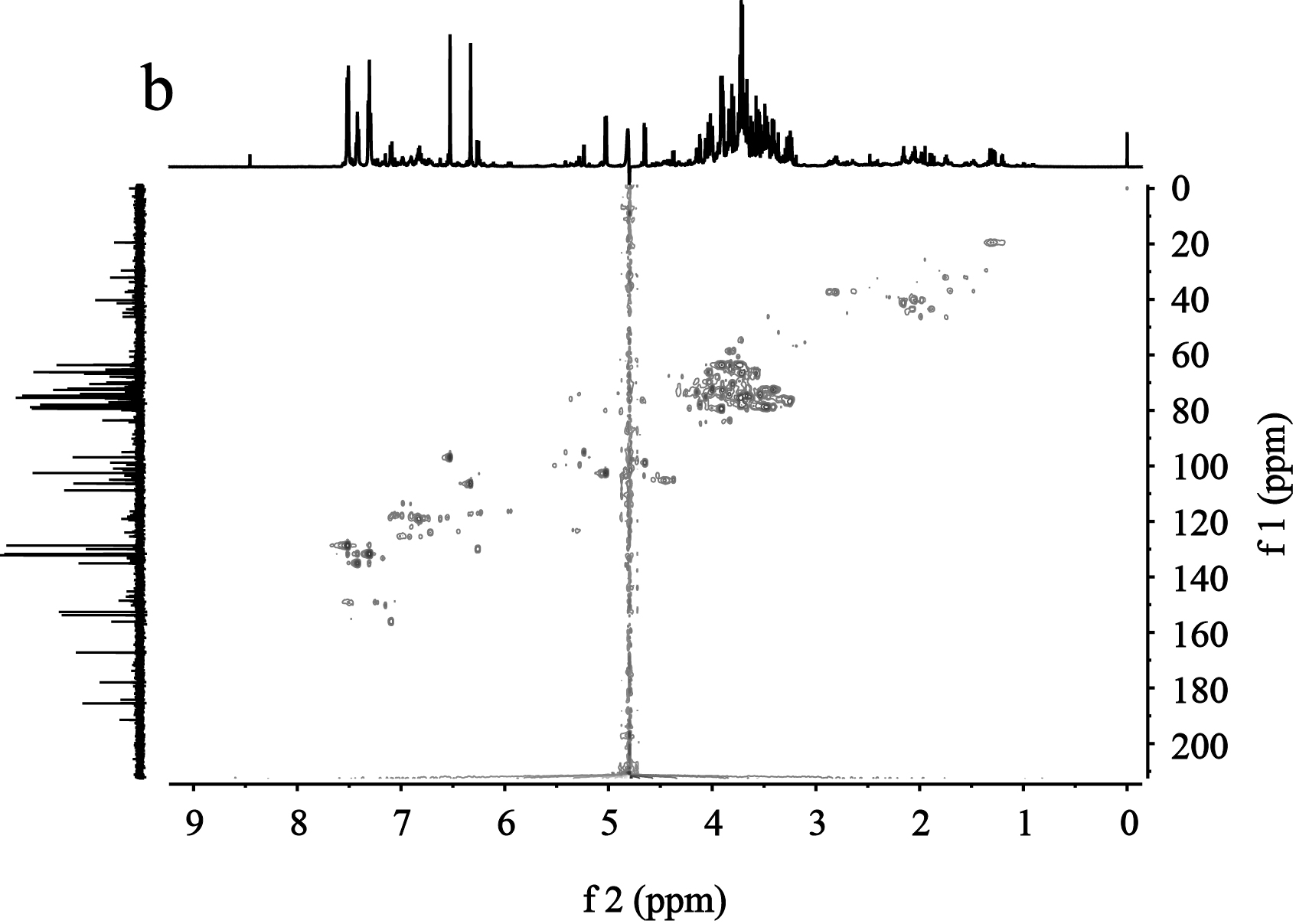


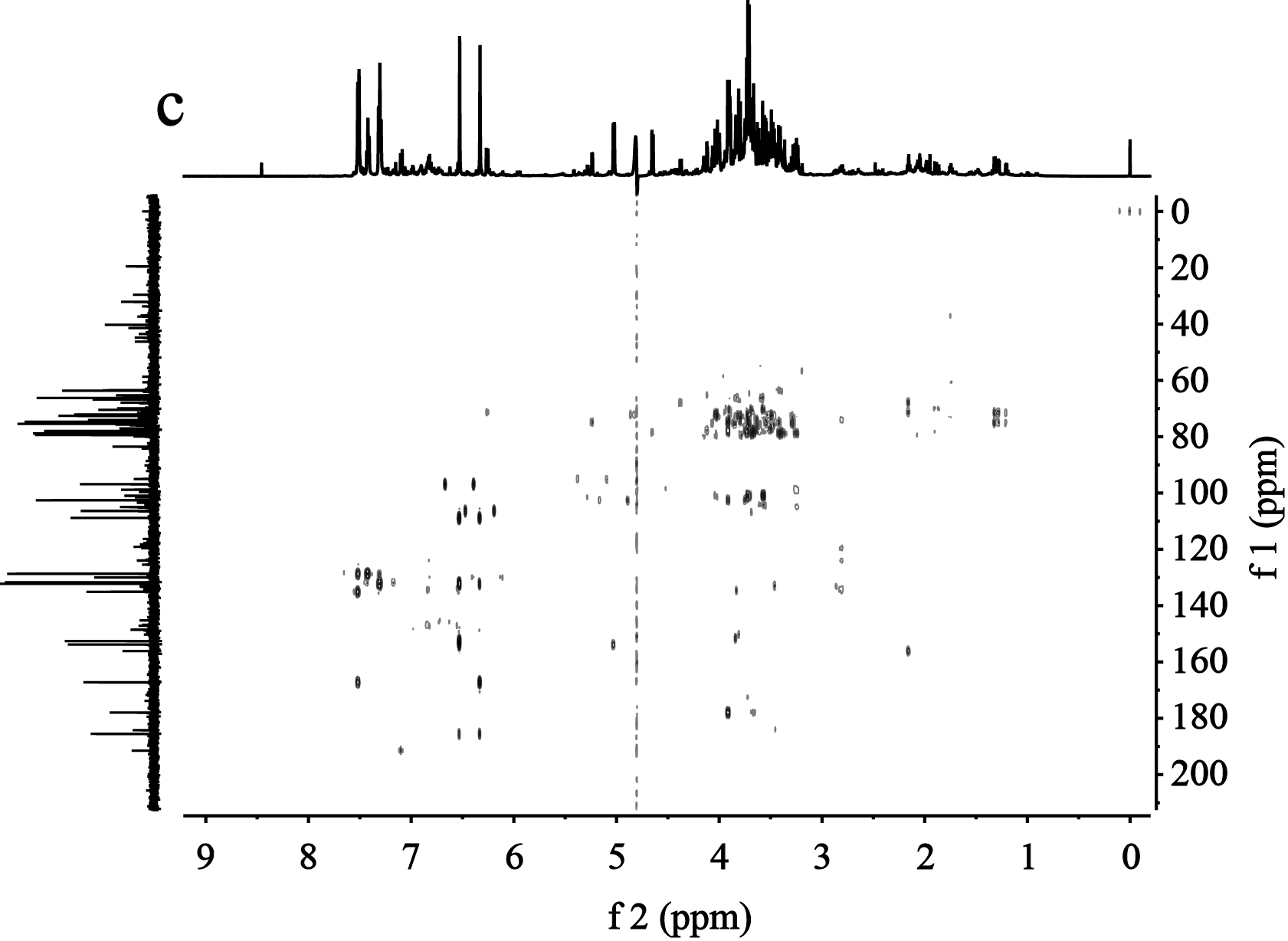


Figure S2: 2D NMR spectra of SHLI (a, ^1^H-^1^H COSY; b, HSQC; c, HMBC).

Table S1: The quantified results of seven primary metabolites in 20 batches of SHLIs (*n* = 3, mg/mL).

| Batches | Valine | Glucose | Fructose | Mannose | Sucrose | Succinic acid | *myo*-Inositol |
| --- | --- | --- | --- | --- | --- | --- | --- |
| B1 | 0.0324 | 1.51 | 0.176 | 3.08 | 0.437 | 0.0410 | 1.36 |
| B2 | 0.0289 | 1.41 | 0.170 | 2.92 | 0.464 | 0.0404 | 1.29 |
| B3 | 0.0410 | 1.82 | 0.187 | 3.44 | 0.454 | 0.0447 | 1.57 |
| B4 | 0.0323 | 1.80 | 0.183 | 3.36 | 0.559 | 0.0380 | 1.41 |
| B5 | 0.0416 | 1.99 | 0.183 | 3.71 | 0.560 | 0.0434 | 1.66 |
| B6 | 0.0313 | 1.15 | 0.159 | 2.75 | 0.302 | 0.0401 | 1.14 |
| B7 | 0.0303 | 1.49 | 0.171 | 3.03 | 0.307 | 0.0369 | 1.18 |
| B8 | 0.0323 | 1.60 | 0.174 | 3.14 | 0.303 | 0.0294 | 1.31 |
| B9 | 0.0314 | 1.54 | 0.200 | 3.20 | 0.292 | 0.0385 | 1.37 |
| B10 | 0.0367 | 1.46 | 0.149 | 3.04 | 0.561 | 0.0362 | 1.37 |
| B11 | 0.0303 | 1.38 | 0.161 | 2.89 | 0.525 | 0.0271 | 1.37 |
| B12 | 0.0311 | 1.23 | 0.131 | 2.53 | 0.548 | 0.0264 | 1.24 |
| B13 | 0.0342 | 1.62 | 0.145 | 3.14 | 0.854 | 0.0257 | 1.53 |
| B14 | 0.0369 | 1.50 | 0.149 | 3.09 | 0.646 | 0.0384 | 1.51 |
| B15 | 0.0386 | 1.77 | 0.174 | 3.54 | 0.532 | 0.0429 | 1.55 |
| B16 | 0.0404 | 1.81 | 0.185 | 3.51 | 0.669 | 0.0382 | 1.65 |
| B17 | 0.0337 | 1.70 | 0.169 | 3.32 | 0.441 | 0.0348 | 1.39 |
| B18 | 0.0288 | 1.24 | 0.130 | 2.67 | 0.642 | 0.0263 | 1.32 |
| B19 | 0.0314 | 1.38 | 0.141 | 3.02 | 0.643 | 0.0320 | 1.37 |
| B20 | 0.0315 | 1.77 | 0.169 | 3.45 | 0.513 | 0.0321 | 1.41 |
